# The importance of the belief that “light” cigarettes are smoother in misperceptions of the harmfulness of “light” cigarettes in the Republic of Korea: a nationally representative cohort study

**DOI:** 10.1186/s12889-015-2472-0

**Published:** 2015-11-07

**Authors:** Annika C. Green, Geoffrey T. Fong, Ron Borland, Anne C. K. Quah, Hong Gwan Seo, Yeol Kim, Tara Elton-Marshall

**Affiliations:** Department of Psychology, University of Waterloo, 200 University Ave West, Waterloo, Ontario N2L 3G1 Canada; School of Public Health and Health Systems, University of Waterloo, Waterloo, Ontario Canada; Ontario Institute for Cancer Research, Toronto, Ontario Canada; The Cancer Council, Melbourne, Victoria Australia; Center for Cancer Prevention and Detection, National Cancer Center, Goyang, Republic of Korea; Division of Cancer Management and Policy, National Cancer Control Institute, National Cancer Center, Goyang, Republic of Korea; Social and Epidemiological Research Department, Centre for Addiction and Mental Health, London, Ontario Canada; Clinical Public Health, Dalla Lana School of Public Health, University of Toronto, Toronto, Canada; School of Health Studies, Western University, London, Canada; Department of Epidemiology and Biostatistics, Western University, London, Canada

**Keywords:** Smoking, Light cigarettes, Packaging and labeling, Public policy, Cigarette design

## Abstract

**Background:**

A number of countries have banned misleading cigarette descriptors such as “light” and “low-tar” as called for by the WHO Framework Convention on Tobacco Control. These laws, however, do not address the underlying cigarette design elements that contribute to misperceptions about harm. This is the first study to examine beliefs about “light” cigarettes among Korean smokers, and the first to identify factors related to cigarette design that are associated with the belief that “light” cigarettes are less harmful.

**Methods:**

We analysed data from Wave 3 of the ITC Korea Survey, a telephone survey of a nationally representative sample of 1,753 adult smokers, conducted October – December 2010. A multinomial logistic regression was used to examine which factors were associated with the belief that “light” cigarettes are less harmful than regular cigarettes.

**Results:**

One quarter (25.0 %) of smokers believed that “light” cigarettes are less harmful than regular cigarettes, 25.8 % believed that smokers of “light” brands take in less tar, and 15.5 % held both of these beliefs. By far the strongest predictor of the erroneous belief that “light” cigarettes are less harmful was the belief that “light” cigarettes are smoother on the throat and chest (*p* < 0.001, OR = 44.8, 95 % CI 23.6–84.9).

**Conclusions:**

The strong association between the belief that “light” cigarettes are smoother on the throat and chest and the belief that “light” cigarettes are less harmful, which is consistent with previous research, provides further evidence of the need to not only ban “light” descriptors, but also prohibit cigarette design and packaging features that contribute to the perception of smoothness.

## Background

Throughout the history of cigarettes, the tobacco industry has responded to increasing evidence of the harms of smoking through innovations in product design and marketing to alleviate the fears of health-concerned smokers [1]. Chief among these innovations has been the “light” cigarette, in which a design element (filter ventilation) is combined with a brand descriptor (“light”) that together conveys the notion that these cigarettes are less harmful [1–4]. Machine testing of “light” cigarettes has yielded reduced levels of tar and nicotine emissions, causing tobacco companies to market them as healthier alternatives to regular cigarettes [1–4].

It is now well-established that “light” cigarette brands are no less harmful than regular brands [1, 2, 4–9]. Evidence has shown that smokers of “light” cigarettes engage in compensatory behaviors to obtain the same amount of nicotine, such as taking deeper and more frequent inhalations, increasing puff velocity, blocking vents with lips or fingers, smoking to a shorter butt length, and smoking more cigarettes per day [6]. Epidemiological studies have shown that there is no difference in lung cancer mortality rates between smokers of “light” and regular brands [7, 8]. Moreover, there is some evidence to suggest that health-concerned smokers switch to “light” brands instead of quitting [3, 4, 9, 10].

Most of the evidence regarding perceptions of “light” cigarettes has come from Western countries; less is known about the prevalence of these beliefs in non-Western countries. A study among Malaysian smokers showed that the prevalence of beliefs that “light” cigarettes are less harmful was comparable to rates found in Western countries (19 % among Malaysian smokers [11] compared to 15 % in Canada, 19 % in the USA, and 16 % in Australia [12]). The same study, however, found much higher rates of misperceptions among Thai smokers with 46 % of smokers believing “light” cigarettes are less harmful [11]. A study conducted in China found even higher rates with 56 % of smokers holding these erroneous beliefs [13].

Importantly, research has indicated that in addition to marketing strategies implemented by the tobacco industry, the sensory experience of smoking “light” cigarettes can influence a smoker’s erroneous belief of reduced harm [2, 3, 9, 13–15]. The additional filter ventilation dilutes tobacco smoke with clean air, giving “light” cigarettes a weaker taste and causing less irritation to the throat and chest when smoked [1–4]. Previous research has shown that this smoothness sensation is associated with the belief of less harmfulness [2, 13–16]. A study that included smokers from Canada, USA, UK, and Australia found that the majority of smokers believed that “light” cigarettes are smoother on the throat and chest than regular cigarettes, and this was strongly correlated with the belief that these cigarettes had lower health risks [2]. In China, this smoothness sensation associated with “light” cigarettes was the factor that was an extremely strong predictor of the belief that “light” cigarettes are less harmful [13]. Recognizing that these associations exist is therefore a critical first step in the development of strategies to counter these false beliefs.

Strategies have been developed to address the problem of false beliefs about “light” cigarettes. Article 11 of the World Health Organization (WHO) Framework Convention on Tobacco Control (FCTC) stipulates that ratifying Parties should adopt and implement effective packaging and labeling measures to ensure that tobacco products are not promoted through the use of false, misleading, or deceptive terms (such as “low-tar”, “light” or “mild”) that are likely to create a wrong impression about its characteristics or health effects, or create the false impression that the tobacco product is less harmful than other products [17]. Plain packaging regulations, which prohibit the use of logos, brand imagery, promotional text and standardize the color, size, format and materials of tobacco packages [18], can further reduce false beliefs about risk by eliminating package design features that have been shown to be associated with notions of smoothness or reduced harm [18–23]. In fact, research has shown that individuals presented with plain packages are less likely to wrongly believe that some cigarette brands are less harmful than others [23]. Australia became the first country to implement a plain packaging law in 2012 [18] and as of October 2015, other countries including Ireland, Britain, France, and Norway are in the process of implementing plain packaging [24]. However, regulations banning product design elements that influence smokers’ sensory perception (e.g., filter ventilation that decreases the harshness of smoking) have not yet been developed, although such regulations would fall within the scope of Article 9 of the FCTC (Product Regulation).

This article reports the first ever study on the beliefs about “light” cigarettes in the Republic of Korea, the eighth largest cigarette market in the world [25]. Given the high rates of smoking (48.3 % of males and 6.3 % of females in 2010 [26]), it is particularly important to document and understand how the deceptive nature of “light” cigarettes influences perceptions of relative harm among Korean smokers. Using data from the International Tobacco Control (ITC) Korea Survey, this article reports the perceptions of relative risk regarding “light” and regular cigarette brands, as well as which factors are independently associated with these beliefs.

## Methods

### Procedure

The data for this study were taken from Wave 3 of the ITC Korea Survey, a nationally representative cohort telephone survey conducted from October to December 2010 (requests for the supporting data for this study may be made through [http://itcproject.org/forms]). To ensure random selection of households, a random-digit dialing method was used within strata defined by 16 geographic areas (9 provinces and 7 metropolitan cities). Allocation of the smoker sample was proportional to the estimated size of the adult population in each stratum. The next birthday method was used to select a respondent in households with multiple smokers. Informed verbal consent was required before interviewing the respondents, all of which were adults aged 19 years and older [27].

Research ethics approval was obtained from the Office of Research at the University of Waterloo, Canada, and the National Cancer Center, Republic of Korea.

### Sample

Survey respondents were ever-smokers aged 19 years and older who had smoked more than 100 cigarettes in their lifetime and had smoked at least once in the past 30 days. A total of 1,753 ever-smokers were surveyed: 1,029 who had participated in previous survey waves, and 724 who were replenished using the same random sampling methods. Questionnaires were first developed in English and then translated to Korean by ITC Korea team members. A complete description of the interview procedure and survey content can be found elsewhere [28]. Only current smokers (*n* = 1,560) were included in the analyses as our study investigates beliefs of less harm among current Korean smokers.

### Measures

#### Beliefs about “light” cigarettes

To assess beliefs about “light” cigarettes, respondents were asked if they strongly agree, agree, neither agree nor disagree, disagree, or strongly disagree with the following statements: “Light cigarettes are less harmful than regular-strength cigarettes”, and “Smokers of light cigarettes take in less tar than smokers of regular-cigarettes”. Responses were coded so that “strongly agree” and “agree” were coded as 1 and the remaining responses were coded as 0. If the respondent refused to answer the question, it was coded as missing and not included in the analyses (this was done for all variables with the exception of income and education, which were categorized as ‘not stated’). These two variables were combined into a single dependent variable of the misperception that “light” cigarettes are less harmful (2 = smokers agree with both statements, 1 = smokers agree with one statement, 0 = smokers disagree with both statements).

#### Demographics and smoking behavior

Demographic measures included: sex, age (18–24, 25–39, 40–54, 55+), income (low: < 30 million KRW (approximately < $26,500 USD), middle: 30 – 60 million KRW (approximately $26,500 USD– $53,000 USD), high: > 60 million KRW (approximately > $53,000 USD)), level of education (low: high school or below, high: more than high school), region, and smoking frequency (daily or non-daily).

#### Health knowledge

To assess knowledge of health effects of smoking, respondents were asked whether smoking cigarettes causes: stroke, impotence, blindness, wrinkling and aging of the skin, peripheral vascular disease, bladder cancer, breast cancer, and heart attack in non-smokers from second-hand smoke (1 = yes, 0 = no/don’t know,). Health knowledge was measured as the sum of all eight responses.

#### Health concerns about smoking

Health concerns about smoking were assessed by asking: “To what extent, if at all, has smoking damaged your health?” (not at all/don’t know, just a little, a fair amount, a great deal); “How worried are you, if at all, that smoking will damage your health in the future?” (not at all, a little worried, moderately worried, very worried); and “Do you consider yourself addicted to cigarettes?” (not at all/don’t know, yes – somewhat, yes – very). Respondents were also asked to rate their health from 1 = poor to 4 = very good.

#### Quitting behavior

To assess quit intentions, respondents were asked “Are you planning to quit smoking?” (not/don’t know, within the next month, within the next 6 months, sometime in the future beyond 6 months). Quitting efficacy was assessed by asking respondents “If you decided to give up smoking completely in the next 6 months, how sure are you that you would succeed? (not at all sure/don’t know, somewhat sure, very sure, and extremely sure).

#### Sensory beliefs about “light” cigarettes

Respondents were asked: “Light cigarettes are smoother on your throat and chest than regular-strength cigarettes”. Responses were coded so that “strongly agree” and “agree” were coded as 1 and remaining responses coded as 0.

#### Statistical analyses

Statistical analyses were conducted using SAS 9.4. With the exception of the sample characteristics (Table 1), all analyses were based on weighted data. A multinomial logistic regression was used to examine which factors were associated to respondents holding one misperception (Model 1) or both misperceptions (Model 2) about “light” cigarettes (models were adjusted for strata).

**Table 1:** Unweighted sample characteristics for smokers of the Wave 3 ITC Korea Survey (*n* = 1560)

		*n*	%
Sex			
	Male	1484	95.1
	Female	76	4.9
Age group (years)			
	18–24	107	6.9
	25–39	382	24.5
	40–54	486	31.1
	55+	585	37.5
Income			
	Low	682	43.7
	Middle	481	30.8
	High	175	11.2
	Don’t know/Not stated	221	14.3
Education			
	Low	881	56.5
	High	650	41.7
	Not stated	28	1.8
Smoking status			
	Daily	1501	96.2
	Non-daily	59	3.8
Cigarettes per day			
	1–10	535	34.4
	11–20	792	50.9
	21–30	149	9.6
	31+	78	5.1

## Results

Table 1 presents the unweighted sample characteristics of smokers in the Wave 3 ITC Korea Survey. The majority of respondents were men (95.1 %), reflecting the gender-related differences in smoking rates. Smoking rates were highest among older age groups and among lower-income groups. Nearly all (96.2 %) respondents reported being daily smokers.

Table 2 presents the weighted percentages of key beliefs. One quarter (25.0 %) of smokers agreed that “light” cigarettes are less harmful than regular strength cigarettes. Similarly, 25.8 % of smokers agreed that smokers of “light” cigarettes take in less tar. These two variables were highly correlated (r_tet_ = 0.71) and were therefore combined into one misperception variable defined by whether smokers agreed with none, one, or both misperceptions. Results showed that over one-third (35.2 %) of Korean smokers held at least one misperception about light cigarettes: 19.7 % of smokers held one of the two misperceptions, and 15.5 % held both misperceptions. In addition, 39.5 % of Korean smokers believed that light cigarettes are smoother on the throat and chest.

**Table 2:** Percentages of key misperceptions about “light” cigarettes

Belief	Agree	
	*n*	%*
“Light” cigarettes are less harmful	392	25.0
Smokers of “light” cigarettes take in less tar	404	25.8
Number of misperceptions held about “light” cigarettes		
*0*	995	64.8
*1*	297	19.7
*2*	247	15.5
“Light” Cigarettes are smoother on the throat and chest	596	39.5

*Belief percentages are weighted

Table 3 presents the results of the weighted multinomial logistic regression performed to determine which factors were independently associated with respondents holding either one misperception (Model 1) or both misperceptions (Model 2) about “light” cigarettes. Out of all of the variables included in the analyses, the only variable that was predictive in both models was the belief that “light” cigarettes are smoother on the throat and chest, and it was a very strong predictor in Model 2 (*p* < 0.001, OR = 44.8, 95 % CI 23.6–84.9). We also found that holding both misperceptions about “light” cigarettes was significantly associated with older age (40–54 years: *p* = 0.03, OR = 2.77, 95 % CI 1.10–7.02; 55+ years: *p* = 0.01, OR = 3.36, 95 % CI 1.31–8.59) and with being “a little worried” about how smoking will damage health (*p* = 0.04, OR = 2.29, 95 % CI 1.06–4.97).

**Table 3:** Weighted multinomial logistic regression of misperceptions about “light” cigarettes

Factor		MODEL 1		MODEL 2	
		1 vs. 0 Misperceptions		2 vs. 0 Misperceptions	
		OR (95 % CI)	*p* Value	OR (95 % CI)	*p* Value
Demographic Variables					
Sex	*Male*	1.50 (0.59 to 3.85)	0.40	0.91 (0.32 to 2.60)	0.86
	*Female*	1.00 (reference)		1.00 (reference)	
Age group (years)	*55+*	1.14 (0.58 to 2.26)	0.70	3.36 (1.31 to 8.59)	0.01*
	*40–54*	1.30 (0.68 to 2.47)	0.43	2.77 (1.10 to 7.02)	0.03*
	*25–39*	1.02 (0.54 to 1.93)	0.96	1.92 (0.75 to 4.96)	0.18
	*18–24*	1.00 (reference)		1.00 (reference)	
Income	*DK/Not stated*	1.20 (0.62 to 2.34)	0.59	1.23 (0.51 to 2.95)	0.64
	*Low*	0.99 (0.57 to 1.74)	0.98	0.74 (0.38 to 1.45)	0.38
	*Middle*	0.85 (0.50 to 1.47)	0.57	0.87 (0.46 to 1.67)	0.68
	*High*	1.00 (reference)		1.00 (reference)	
Education	*Not stated*	0.72 (0.14 to 3.64)	0.69	1.06 (0.18 to 6.34)	0.95
	*Low*	1.26 (0.86 to 1.85)	0.23	1.05 (0.66 to 1.67)	0.83
	*High*	1.00 (reference)		1.00 (reference)	
Smoking Behavior					
Smoking status	*Daily*	1.00 (0.41 to 2.46)	0.99	1.06 (0.26 to 4.39)	0.94
	*Non-daily*	1.00 (reference)		1.00 (reference)	
Daily consumption	*Increments of 10*	1.01 (0.82 to 1.26)†	0.90	0.86 (0.64 to 1.15)†	0.31
Health Knowledge	*1–8*	0.99 (0.92 to 1.07)†	0.79	0.95 (0.87 to 1.04)†	0.29
Health Concern					
Worried smoking has damaged health	*A lot*	0.97 (0.50 to 1.87)	0.92	0.82 (0.36 to 1.83)	0.62
	*A fair amount*	1.18 (0.63 to 2.20)	0.61	1.05 (0.49 to 2.29)	0.89
	*A little*	1.12 (0.69 to 1.81)	0.65	0.94 (0.53 to 1.69)	0.84
	*Not at all*	1.00 (reference)		1.00 (reference)	
Worried smoking will damage health	*Very worried*	1.41 (0.65 to 3.04)	0.38	1.13 (0.45 to 2.82)	0.80
	*Moderately worried*	1.16 (0.55 to 2.41)	0.70	1.65 (0.70 to 3.86)	0.25
	*A little worried*	1.08 (0.57 to 2.05)	0.81	2.29 (1.06 to 4.97)	0.04*
	*Not at all*	1.00 (reference)		1.00 (reference)	
Rate your health	*Poor – very good*	0.86 (0.68 to 1.08)†	0.20	0.95 (0.71 to 1.26)†	0.70
Perceived addiction	*Very addicted*	0.91 (0.48 to 1.72)	0.78	1.34 (0.62 to 2.90)	0.47
	*Somewhat addicted*	1.34 (0.73 to 2.47)	0.34	1.42 (0.68 to 3.00)	0.36
	*Not at all*	1.00 (reference)		1.00 (reference)	
Quitting					
Quit Intentions	*Within 1 month*	0.82 (0.41 to 1.66)	0.58	1.24 (0.54 to 2.84)	0.61
	*Within 6 months*	0.94 (0.56 to 1.59)	0.82	1.52 (0.82 to 2.82)	0.19
	*Beyond 6 months*	0.98 (0.64 to 1.51)	0.93	1.20 (0.68 to 2.11)	0.53
	*No intentions*	1.00 (reference)		1.00 (reference)	
Quit efficacy	*Extremely sure*	0.71 (0.34 to 1.48)	0.35	1.69 (0.65 to 4.36)	0.28
	*Very sure*	0.95 (0.48 to 1.85)	0.87	1.52 (0.68 to 3.38)	0.31
	*Moderately sure*	1.11 (0.65 to 1.88)	0.71	1.63 (0.81 to 3.29)	0.17
	*Slightly sure*	1.00 (0.60 to 1.67)	1.00	1.37 (0.70 to 2.68)	0.36
	*Not at all sure*	1.00 (reference)		1.00 (reference)	
Smoothness beliefs					
“Lights” are smoother	*Agree*	3.86 (2.72 to 5.47)	<.001*	44.8 (23.6 to 84.9)	<.001*
	*Disagree*	1.00 (reference)		1.00 (reference)	

Logistic regression controlled for region †Continuous variable *Statistically significant

Table 4 presents the weighted cross tabulation of the number of misperceptions about “light” cigarettes and those who agree that “light” cigarettes are smoother on the throat and chest that illustrates the very strong relation between the two variables. Among smokers who believed that “light” cigarettes are smoother on the throat and chest, over one third (36.3 %) believed that “light” cigarettes are less harmful and deliver less tar. In contrast, among smokers who did *not* believe that “light” cigarettes are smoother on the throat and chest, only 2.1 % believed in both of these misperceptions.

**Table 4:** Cross tabulation of misperceptions about “light” cigarettes and the belief that “light” cigarettes are smoother

		Number of misperceptions held about “light cigarettes			
“Light” cigarettes are smoother on the throat and chest		0	1	2	Total
	Agree	37.2 %	26.5 %	36.3 %	100 %
	Disagree	82.6 %	15.3 %	2.1 %	100 %

Percentages are weighted

## Discussion

Over one-third (35.2 %) of smokers in the Republic of Korea hold at least one misperception that “light” cigarettes offer health benefits. One quarter (25.0 %) of smokers in the Republic of Korea wrongly believe that “light” cigarettes are less harmful. These levels of incorrect beliefs are higher than those found in Canada (14.8 %), the USA (19.0 %), and Australia (15.5 %) [12]. Our study also showed that one quarter (25.8 %) of Korean smokers believe that smokers of “light” cigarettes take in less tar, and that 15.5 % of Korean smokers hold both misperceptions.

Compared to rates of misperceptions on the harmfulness of “light” cigarettes found in many Western countries, the slightly higher rates among Korean smokers may be a reflection of the lenient regulations regarding the advertising and packaging of tobacco products in the Republic of Korea. With tobacco advertising prohibited in mass media, the primary channels of marketing communication utilized by tobacco companies are retail merchandizing (product advertising at point-of-sales), print media (such as magazines with the exception of those intended for women and youth), and cigarette packaging [29]. Packaging and labeling in particular has been a key strategy implemented by tobacco companies to communicate notions of harm reduction or health reassurance to Korean smokers [30, 31]. According to market research conducted by the tobacco industry, “package design can make an inferential statement that, in relative terms, the brand is a more clean and healthy alternative” [31]. Although the Korean government banned misleading descriptors including “light” and “mild” on cigarette packages in January 2015 [32], plain packaging is an important and necessary step in reducing the misperception that some cigarette brands are less harmful than others.

In line with previous research [2, 3, 9, 13–16], we also found that the belief that “light” cigarettes were smoother on the throat and chest was significantly associated with the belief that “light” cigarettes are less harmful. In fact, smokers who believed that “light” cigarettes are smoother on the throat and chest had 45 times greater odds of also believing that “light” cigarettes are less harmful and deliver less tar (36 % vs. 2 %). This extremely high association suggests that banning misleading descriptors or other package features that are evocative of lightness may not be enough. Ultimately, the strong association between the smoothness sensation and the belief of reduced harm illustrate the need to implement regulations to ban cigarette design features that reinforce these incorrect beliefs by decreasing the harshness of tobacco smoke, such as ventilated filters or flavorings such as menthol [15].

With respect to limitations of this study, the survey did not ask respondents to identify the strength of the cigarette that they currently smoked (“light” or “low-tar”). We therefore do not know whether smokers of “light” brands are more likely to believe that “light” cigarettes are less harmful than regular cigarettes, although we would expect that this would be the case based on previous research [2, 4, 9, 13, 14, 16]. We also do not know whether smokers’ beliefs that “light” cigarettes are smoother than regular cigarettes is based on their own personal experience, as this belief can be derived from other sources such as cigarette packaging and descriptors, even for non-smokers and smokers of other brands [21].

## Conclusions

The ban on misleading descriptors on cigarette packages that the Korean government implemented in January 2015 is an important first step in addressing false beliefs that some cigarette brands are less harmful than others. However, prohibiting the use of misleading terms is unlikely to be sufficient in preventing these beliefs because it only addresses marketing strategies employed by the tobacco industry, while failing to address the cigarette design elements that create these misperceptions. Given that the sensory experience of smoking “light” cigarette brands has been shown to lead to beliefs of reduced harm, it is important to remove the cigarette design elements that cause the sensation of reduced strength of smoke [3, 15]. This can largely be achieved by banning filter ventilation [5], which is also supported by the conclusion of the 2014 US Surgeon General’s report [33] that asserted that cigarettes may have become even more harmful, and that filter venting is a prime suspect for this trend.

Ultimately, the introduction of plain packaging, banning “light” and “low-tar” descriptors, and the elimination of cigarette design elements that have been shown to reduce the harshness of smoking may be what is necessary to counteract a strategy that has been used by the tobacco industry to perpetuate misperceptions that some types of cigarettes are less harmful.
